# Establishing a Porcine Eye Model for Manual Sub-Bowman Layer Photorefractive Keratomileusis

**DOI:** 10.1155/2020/9834760

**Published:** 2020-07-14

**Authors:** Mingxia Tian, Ping Ma, Guoying Mu, Lijing Chen, Jie Feng

**Affiliations:** ^1^Department of Ophthalmology, Jining No. 1 People's Hospital Affiliated to Jining Medical College, No. 6 Jiankang Road, Jining, Shandong, China; ^2^Department of Ophthalmology, Shandong Provincial Hospital Affiliated to Shandong University, No. 324 Jingwuweiqi Road, Jinan, Shandong, China

## Abstract

**Objective:**

To establish a porcine eye model for manual sub-Bowman layer photorefractive keratomileusis (SBPRK), which is a reformed surface ablation refractive surgery that results in preserving the corneal Bowman layer (BL).

**Methods:**

The SBPRK group consisted of eleven eyes of 8 healthy pigs with BL flaps by mechanical technique followed by laser ablation. Regarding the remaining 5 eyes, 3 random eyes had transepithelium photorefractive keratectomy (TransPRK) (the TransPRK group), while the other 2 eyes were untreated (the blank control group). All the pigs were followed up for 8 weeks. Slit-lamp biomicroscopy and optical coherence tomography (OCT) were examined before the surgeries and at 1 week, 4 weeks, and 8 weeks after the surgeries.

**Results:**

In a few days after the surgery, 3 eyes of the SBPRK group were excluded from the study because of poor healing of the corneal flaps. At the 1^st^ postoperative week, one eye had an irregular defect of about 3 mm in the central corneal epithelium area; the cornea of the other 7 eyes had just light edema with intact epithelium just like the cornea of the TransPRK group. At the 4^th^ week, in the SBPRK group, the cornea was slightly hazy (haze stage 1). While in the TransPRK group, the cornea was hazier (haze stage 2). At the 8^th^ week, in the SBPRK group, both corneas were almost transparent, and the edges of the BL flaps could not be clearly seen. Meanwhile, in the TransPRK group, the corneal haze became lighter and thinner. OCT showed that, in the SBPRK group, there was high reflection in the BL layer, and it was obvious at 1 week postoperation, decreased at 4 weeks, and calmed down at 8 weeks. However, in the TransPRK group, the high reflection diffused in the anterior corneal stroma at 1 week postoperation, enhanced at 4 weeks, and weakened at 8 weeks.

**Conclusions:**

Preserving the BL while conducting surface refractive surgery may result in less haze than TransPRK. However, further study is still needed, and this technique still requires refining until it becomes a standard clinical procedure.

## 1. Introduction

Corneal laser refractive surgeries have become common for treating refractive errors such as myopia, hyperopia, and astigmatism. In 1987, photorefractive keratectomy (PRK), a surface ablation technique, by Dr. Theo Seiler, became the first excimer laser refractive procedure ever performed [1]. After more than thirty years, many procedures have been developed for correcting ametropia. Such procedures are usually divided into two categories: the surface ablation surgeries and the stromal ablation surgeries. For surface ablation surgeries, such as PRK, laser-assisted subepithelium keratectomy (LASEK), and transepithelium photorefractive keratectomy (TransPRK), the laser ablates just under the corneal epithelium or with the corneal epithelium. The surface ablation surgeries are suitable for low to moderate myopia, as they may result in a corneal haze, but they are devoid of flap-related complications. For stromal ablation surgeries, such as laser-assisted in situ keratomileusis (LASIK), sub-Bowman keratomileusis (SBK), femtosecond laser-assisted LASIK (FS-LASIK), femtosecond lenticule extraction (FLEx), and small incision lenticule extraction (SMILE), the site of the laser ablation lies inside the stroma of the cornea. The stromal surgeries are suitable for low to high myopia with fast vision recovery and little painful complaints.

Although refractive surgeries could decrease the corneal biomechanical strength, surface ablation surgeries have better outcomes. There are several indices to describe the corneal biomechanics. Hashemi et al. [2], using the Corvis ST ocular biomechanical metrics, found that the corneal biomechanics changed more after FS-LASIK than after PRK and mitomycin C in high myopia patients. While Hwang et al. [3] found that both LASIK and PRK decreased the corneal hysteresis (CH) and a corneal resistance factor (CRF) from baseline, these increased to a similar level by 12 months. Guo et al. [4] analyzed 22 studies regarding corneal biomechanics using the ocular response analyzer (ORA), which indicated that SMILE was superior to FS-LASIK and LASIK in preserving corneal biomechanical strength after surgery. They also found that although no significant difference was found, the PRK/LASEK group had better outcomes than SMILE.

In surface ablation procedures, the basal epithelial membrane and the Bowman layer (BL) are ablated along with the stromal tissue. During laser ablation, corneal keratocytes are damaged and undergo apoptosis and necrosis, activating and releasing transforming growth factors (TGFs), which have direct access to the stromal interface. These inflammatory mediators then generate myofibroblasts that proliferate to repair the stroma. Structural changes from this process lead to the disruption of collagen fibrils in the cornea from the deposition of extracellular matrix components, which eventually manifest as corneal haze [5]. Corneal haze is the clouding of the cornea and is a rare but vision-threatening adverse outcome of laser ablation refractive surgery, especially in eyes with high myopia and astigmatism [6]. Haze presents as a white punctate or reticular opacity in the anterior part of the cornea and generally arises 2 months (early haze) or 4 months (late haze) after surface refractive surgery [7].

The absence of the barrier between the epithelium and the stroma might result in haze occurring. Lie et al. performed the first BL transplantation surgery in 2010 to eliminate and prevent the recurrence of subepithelial haze after PRK [8]. BL might increase corneal biomechanism too, and it has been suggested that the BL may be the strongest biomechanical part of the human cornea [9]. BL transplantation allows patients to maintain acceptable visual acuity with glasses or contact lenses and may postpone or prevent the need for penetrating keratoplasty (PK) or deep anterior lamellar keratoplasty (DALK) [10, 11]. BL transplantation could be a promising treatment with a 5-year estimated success rate of 84% regarding advanced keratoconus and corneal ectasia after refractive surgeries, which are too steep or too thin for ultraviolet corneal crosslinking or intracorneal ring segments [10, 11].

So, how about preserving the BL when the surface surgeries are done? In this study, we established a porcine surface ablation eye model with the BL preserved, which we have termed sub-BL photorefractive keratectomy (SBPRK), and then, the corneal recovery was observed.

## 2. Methods

Sixteen eyes of eight healthy two-month-old domestic pigs weighing about 10 kg were concluded in the study. Eleven eyes in the experimental group had SBPRK, 3 eyes in the control group had TransPRK, and 2 eyes without any treatment became the blank controls.

All surgeries were conducted under general anesthesia. The animals were injected intramuscularly with xylazine hydrochloride 0.05 g/kg (Huamu Animal Health Products Co., Ltd.), followed by 0.4% propofol intravenously injection (Fresenius Kabi Austria GmbH) for anesthesia maintenance. After well anesthetized, the SBPRK surgery was performed with the following procedures. Eyelids were opened with an eye speculum; the cornea was immersed with a 20% alcohol solution in an alcohol cone with 8.5 mm diameter for 20 seconds and then irrigated thoroughly with sodium lactate Ringer's injection (Cisen Pharmaceutical Co., Ltd, Jining, China). The corneal epithelium was scraped by a corneal scraper, and the cornea was stained with 0.06% trypan blue resolution (Solarbio Life Sciences, Beijing) for 2 minutes and then irrigated with a sodium lactate Ringer's injection again. A 30-gauge needle was used to incise the BL along the round notch, peripherally. The peripheral BL could be lifted and grasped with microscopic toothless forceps. It was carefully peeled free from the underlying stroma, with an 8.5 mm diameter BL flap obtained, leaving a hinge at the 12 o'clock position, followed by −9 D excimer laser ablation (optic zone 6.50 mm, time 36 s, MAX depth 137 micrometers (*μ*m)) on the stromal bed by Amaris 500E (Schwind Eye-Tech-Solutions, GmbH, Kleinostheim, Germany), and repositioned the BL flap. After tobramycin dexamethasone eye drops were administered, bandage contact lenses (PureVision, Bausch & Lomb Incorporated) were placed after the operations.

The 3 eyes in the TransPRK control group were ablated of −9 D with Amaris 500E aberration-free aspherical optimized profile (optic zone 6.50 mm, time 72 s, MAX depth 195 *μ*m). After tobramycin dexamethasone eye drops were administered, bandage contact lenses (PureVision, Bausch & Lomb Incorporated) were placed after the operations.

All the eyes were examined under general anesthesia with a slit-lamp bioscope and optical coherence tomography (OCT, Carl Zeiss Meditec, Inc., Dublin) before the operation and 1 week, 4 weeks, and 8 weeks after the operations. Levofloxacin eye drops were used for one week, as well as three days before the operations. Tobramycin dexamethasone eye drops and lubricants were administered three times a day postoperatively.

The pigs were forbidden diet 8 hours before anesthesia and then fed normally 4 hours after anesthesia, and they were kept in a clean hog lot which had plenty of sunshine during the observation period. Euthanasia was performed on all the animals with narcotic overdose after finishing all the observations.

Corneal haze was observed with slit-lamp microscopy and graded on a predetermined scale of 0–4, according to the density of opacity. Grade 0 is totally clear cornea, and grade 4 is severe opacity that completely obscured the details of the intraocular structures. According to this grading, cornea with a haze grade of 0 or 0.5 was considered clear [12].

The study was approved by the ethics committee of Jining No. 1 People's hospital affiliated to Jining Medical College.

## 3. Results

**Figure 1:**
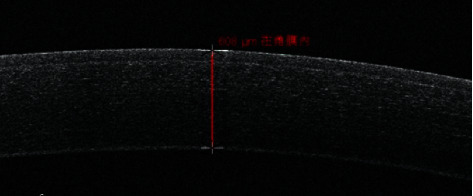
The normal porcine corneal OCT image was similar to that of human being's cornea, and the corneal central thickness was 608 *μ*m.

**Figure 2:**
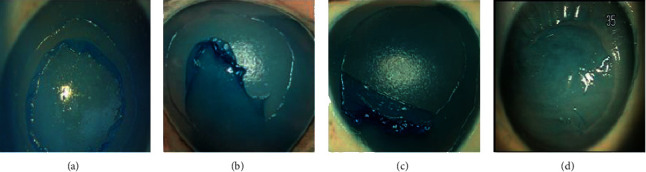
Making a BL flap in a porcine eye. (a) The margin of the BL flap is lifted. (b) The BL flap is half-peeled. (c) The free BL flap with a hinge. (d) Replacement of the BL flap.

The normal porcine cornea was transparent under slit-lamp biomicroscope. Under OCT scan, the structure of the porcine cornea was similar to that of human being's cornea (Figure 1). The central corneal thickness is 573 ± 34.9 *μ*m (*n* = 16). The procedure of making a BL flap is shown in Figure 2.

All the bandage contact lenses had fallen out 2–6 days after the operations. Of the 11 eyes in the experimental group, 3 eyes were excluded from the study because of the bandage lenses prematurely dropping out, which resulted in poor healing of the corneal flaps and inflammation, which eventually led to stromal vascularization. The cornea of the blank control group was transparent during the observation.

**Figure 3:**
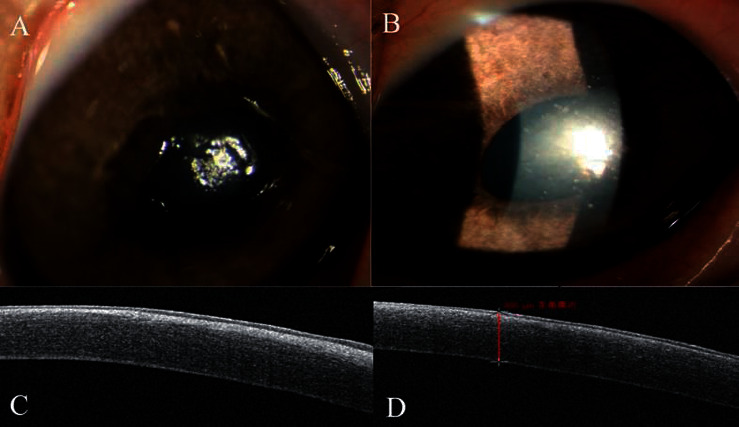
At the 1^st^ postoperative week, (a) in the SBPRK group, the cornea of one of the eyes had light edema, and the epithelium had an irregular defect about 3 mm in the central area, while the BL was well-positioned. (b) In the TransPRK group, the cornea was slightly edematous with nearly intact epithelium. (c) OCT showed a high reflection layer under the epithelium layer in the SBPRK group. (d) The cornea of the TransPRK group had a high reflection obscure layer under the epithelium and some high reflection materials in the stroma.

At the 1^st^ postoperative week, in the SBPRK group, 7 eyes had just light edema with intact epithelium, while the cornea of the remaining one eye had an irregular defect about 3 mm in the central corneal epithelium area with the BL well-positioned. OCT showed high reflection under the epithelium layer (Figures 3(a) and 3(c)). In the TransPRK group, the cornea of the 3 eyes was slightly edematous with nearly intact epithelium, and OCT showed a high reflection obscure layer under the epithelium and some high reflection materials in the stroma (Figures 3(b) and 3(d)).

**Figure 4:**
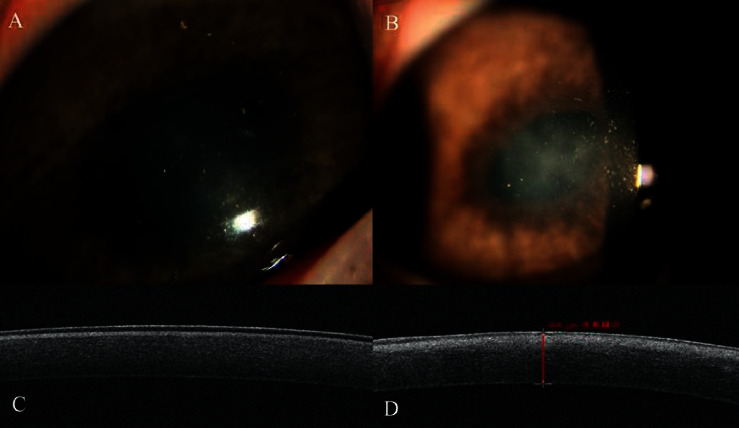
At the 4^th^ postoperative week, (a) in the SBPRK group, the cornea was slightly hazy (haze stage 1) in the center of the cornea. (b) In the TransPRK group, the cornea was hazier (haze stage 2) in the center of the cornea. (c) OCT showed that the cornea of the SBPRK group had a thin high reflection layer just under the epithelium. (d) In the TransPRK group, the corneal thickness was 448 *μ*m and had diffused high reflection in the anterior stroma.

At the 4^th^ postoperative week, in the SBPRK group, the cornea of the 8 eyes was slightly hazy (haze 1 stage) in the center of the cornea (Figure 4(a)). In the TransPRK group, the cornea was hazier (haze stage 2) in the center of the cornea (Figure 4(b)). OCT showed that the cornea of the SBPRK group had a thin high reflection layer just under the epithelium, while the cornea of the TransPRK group had diffused high reflection in the anterior stroma (Figures 4(c) and 4(d)).

**Figure 5:**
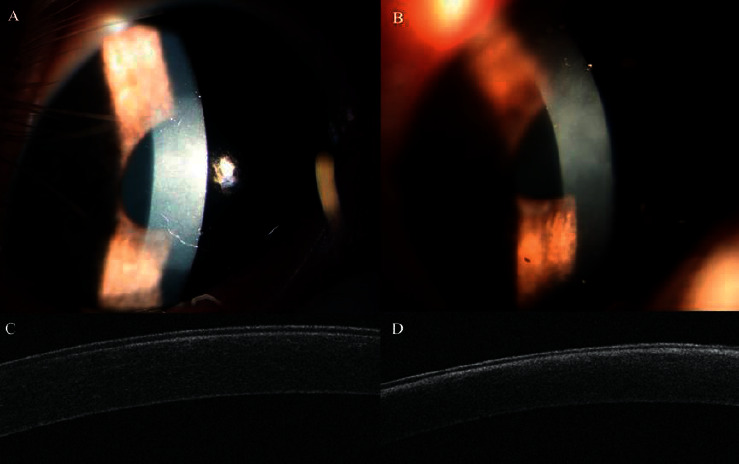
At the 8^th^ postoperative week, (a) the cornea in the SBPRK group was almost transparent. (b) The corneal haze became lighter and thinner in the TransPRK group. (c) OCT showed a nearly normal cornea in the SBPRK group. (d) The corneal high reflection became weaker in the TransPRK group.

At the 8^th^ postoperative week, the cornea in the SBPRK group was almost transparent, and the edges of the BL flaps could hardly be seen (Figure 5(a)). In the TransPRK group, the corneal haze had become lighter and thinner (Figure 5(b)). In the SBPRK group, OCT showed a nearly normal cornea, and in the TransPRK group, the corneal high reflection had weakened compared with that of the 4^th^ postoperative week (Figures 5(c) and 5(d)).

## 4. Discussion

In the study, we established a porcine eye model of manual sub-BL photorefractive keratectomy for the first time, to our knowledge. The results indicated that the SBPRK eyes had less haze compared to the TransPRK eyes. The primary cause is probably that the SBPRK procedure preserved the BL and maintained corneal structures. While TransPRK entails the removal of the epithelium with the underlying BL, as well as a variable part of the anterior stroma, depending on the degree of correction needed, using an excimer laser. This results in the death of keratocytes, activation of the stromal cells outside of the ablation zone, infiltration of the immune cells, and myofibroblast formation. Haze has been observed to occur in all patients within a month postoperatively, then mostly subsides [13]. Korkmaz et al. compared the corneal haze after transepithelial PRK and LASEK and found that at 1 month, higher haze grades and grey scale values in confocal microscopy were noted in transepithelial PRK-treated eyes compared to LASEK-treated eyes. A greater reduction in keratocyte density was observed under confocal microscopy after transepithelial PRK at 1–3 months. These results implied a more intense wound healing response after transepithelial PRK compared to LASEK [14]. But compared with traditional PRK, transepithelial PRK has been seen to result in significantly less haze [15, 16].

The etiology and mechanism of corneal haze formation may be complex, involving TGF-b induced myofibroblasts and excessive ECM production during stromal remodeling [17]. Kumar et al. studied the intraoperative epithelium with postoperative haze after refractive surgeries and revealed a series of novel molecular factors in the cornea associated with corneal haze after refractive surgery such as PREX1, WNT3A, SOX17, GABRA1, and PXDN. They found that, among them, PREX1 was significantly upregulated in haze predisposed subjects [18]. A meta-analysis showed that PRK and LASEK had no significant differences in terms of haze, while epi-LASIK and TransPRK would result in better outcomes [19]. TGF-1 levels positively correlated with the degree of haze formation. TGF-1 is released by the lacrimal gland into the tear film after corneal epithelial injury. Its levels were less following epi-LASIK and TransPRK than after PRK and LASEK [20, 21].

In the study, the corneal epithelium of one of the SBPRK eyes had delayed healing, while the cornea of the TransPRK group all recovered well in the seventh postoperative days. This might be due to that the diameter of scraped epithelium in SBPRK was larger than the epithelial defect in TransPRK. The epithelium was ablated by laser in one step along with the stromal lenses in a TransPRK process, and the ablation diameter was less than 8.5 mm in the manual surgical process. But the BL was well positioned after surgery under bandage lenses, and this made the very thin flap laser surgery feasible. Indeed, there was the probability of epithelial ingrowth theoretically because the BL was lifted and replaced in the surgery. But it might be minor because of the application of bandage lenses.

In the study, OCT scan showed that the cornea after both surgeries took on high reflection in the stroma. The high reflection probably composed of apoptotic keratocytes, inflammatory factors, and myofibroblasts. This meant that both the surgeries could cause an inflammatory reaction in the cornea. In the SBPRK group, their action was in the BL layer, and it was obvious at the1 week postoperation, decreased at 4 weeks, and calmed down at 8 weeks. However, in the TransPRK cornea, the reaction was diffused in the anterior stroma at the 1 week postoperation, enhanced at 4 weeks, and weakened at 8 weeks.

In the study, the BL flaps were made manually. Preparing the BL is not so easy in actuality. It is laborious and technically demanding, with a failure rate of about 30%, which may constrain the technique being commonly used [22]. Making the BL by a femtosecond laser may be easier and quicker. Parker et al. compared the BL grafts made with a femtosecond laser and manually. They found that the mean flap thickness was thinner in the manual group (11.7 ± 1.6 *μ*m) than in the femtosecond group (37 ± 8.6 *μ*m). The femtosecond group had a smoother posterior surface observed using transmission electron microscopy [23]. Moreover, the smoothness of the stromal surface has been associated with the formation of corneal haze [24]. Therefore, femto-LASIK probably has more advantages than a manual technique if it could make a thinner flap.

Meanwhile, manual stripping of the BL flap resulted in a thinner layer of stromal tissue, which was similar to the thin flap created using femtosecond laser LASIK or SBK. It has been reported that after thin flap femto-LASIK laser surgery, more haze was formed than after conventional microkeratome LASIK [25, 26]. As the flaps were cut close to the BL, the basement membrane of the corneal epithelium might have been damaged. Defects in the BL allow contact between the proinflammatory epithelium-derived cytokines such as TGF and the corneal stroma [27], but the haze disappeared quickly [25, 26]. This was similar to our results.

The study does have some limitations. The bandage contact lenses in 8 of the experimental group eyes fell out on the fifth to sixth postoperative days, which resulted in the corneal flaps healing well. Those that fell out before this had a wrinkled BL flap, that became inflamed, which resulted in corneal pannus. The BL flap was very thin and easy to wrinkle despite wearing the bandage lenses. Perhaps, the flap should be sutured at the very beginning to improve the success rate in animal models. Among the experimental animals, pigs have cornea with a BL similar to humans [28], but it is too big to be conveniently observed, which made certain examinations such as confocal microscopy challenging to do. There may be better ways to do this, and further study is needed.

## 5. Conclusions

Conducting surface refractive surgery while preserving the BL remaining probably could result in less haze than TransPRK. However, further study is still needed, and technique still requires refining until it becomes a standard clinical procedure due to issues with the manual technique and corneal healing.

## Data Availability

The data used to support the findings of this study are included within the article.
